# An innovative approach to using an intensive field course to build scientific and professional skills

**DOI:** 10.1002/ece3.9446

**Published:** 2022-10-22

**Authors:** Adrienne B. Nicotra, Sonya R. Geange, Nur H. A. Bahar, Hannah Carle, Alexandra Catling, Andres Garcia, Rosalie J. Harris, Megan L. Head, Marvin Jin, Michael R. Whitehead, Hannah Zurcher, Elizabeth A. Beckmann

**Affiliations:** ^1^ Research School of Biology The Australian National University Canberra Australian Capital Territory Australia; ^2^ Department of Biological Sciences University of Bergen Bergen Norway; ^3^ Bjerknes Center for Climate Research Bergen Norway; ^4^ School of Biological Sciences The University of Queensland Brisbane Queensland Australia; ^5^ School of BioSciences The University of Melbourne Parkville Victoria Australia

**Keywords:** cognitive apprenticeship, field course, fieldwork, functional ecology, researcher identity, university teaching

## Abstract

This paper reports on the design and evaluation of Field Studies in Functional Ecology (FSFE), a two‐week intensive residential field course that enables students to master core content in functional ecology alongside skills that facilitate their transition from “student” to “scientist.” We provide an overview of the course structure, showing how the constituent elements have been designed and refined over successive iterations of the course. We detail how FSFE students: (1) Work closely with discipline specialists to develop a small group project that tests an hypothesis to answer a genuine scientific question in the field; (2) Learn critical skills of data management and communication; and (3) Analyze, interpret, and present their results in the format of a scientific symposium. This process is repeated in an iterative “cognitive apprenticeship” model, supported by a series of workshops that name and explicitly instruct the students in “hard” and “soft” skills (e.g., statistics and teamwork, respectively) critically relevant for research and other careers. FSFE students develop a coherent and nuanced understanding of how to approach and execute ecological studies. The sophisticated knowledge and ecological research skills that they develop during the course is demonstrated through high‐quality presentations and peer‐reviewed publications in an open‐access, student‐led journal. We outline our course structure and evaluate its efficacy to show how this novel combination of field course elements allows students to gain maximum value from their educational journey, and to develop cognitive, affective, and reflective tools to help apply their skills as scientists.

## Graphical abstract

Using extensive evaluation data, we show how field studies support students to develop a coherent and nuanced understanding of how to approach and execute ecological studies. We illustrate how field course elements can be used to provide cognitive, affective, and reflective tools that allow students to gain maximum value from their educational journey, and the confidence to consider research in their future careers.

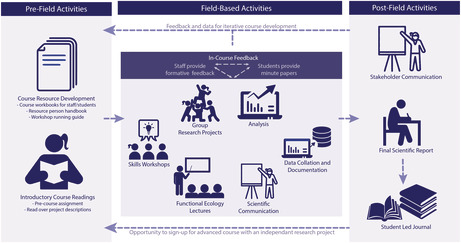

## 1 INTRODUCTION

Since the 1990s, the logistical, resourcing, and equity challenges of residential ecology field courses have seen such courses become increasingly rare become increasingly rare in university teaching (Boyle et al., 2007; Cotgreave, 1996). Yet, for university students in ecology, well‐structured hands‐on activities uniquely build practical research skills (Jackson, 2016) while providing experiences of the excitement and frustration of hypothesis‐driven research, data collection and analysis, and collaboration (Abrams et al., 2018; Beckmann et al., 2015; Estavillo et al., 2014; Pedaste et al., 2015). Indeed, field courses are associated with higher self‐efficacy gains, higher college graduation rates, higher retention in the ecology and evolutionary biology major, and higher Grade Point Averages at graduation compared to lecture‐based courses (Beltran et al., 2020; Scott et al., 2012). The skills attained in field courses also translate to increased graduate employability (Mauchline et al., 2013; Peacock & Bacon, 2018). Studying in the field also helps students understand that nature is incredibly complex, integrated and interdependent, and requires interdisciplinary thinking (Behrendt & Franklin, 2014; Durrant & Hartman, 2015; Geange et al., 2021). To maximize the value of learning and the return on investment, therefore, a best practice field course needs to be cost‐effective and efficient and provide multiple benefits for both students and teaching staff that extend beyond new discipline knowledge to broader career‐enhancing skills.

In this paper, we describe our experiences and evaluations of several years of the Field Studies in Functional Ecology (FSFE) course. During 2 weeks in the field, coached by experts and peers and supported by appropriately scheduled skills workshops, our students iteratively design and implement customized research projects or “field problems.” Students work in small groups to identify their own research questions, design a research protocol, collect and analyze data, and present their findings and interpretations to the group and external stakeholders. Uniquely, student groups develop “rapid prototypes” of a project before swapping it with a new group for refinement and expansion, and after the course can publish their work in an open‐access, student‐edited journal, closing the research loop through first‐hand exposure to scientific publishing.

We designed FSFE to maximize both the value of learning and the return on investment by enabling students to master core content in functional ecology alongside broader employability skills. Boyer (1990), in his seminal work on scholarship, argued that knowledge is acquired through research, synthesis, practice, and teaching. These are all foundational principles in FSFE's design, not only in the activities provided *for* our students, but also those provided *with* and *by* them, in line with principles of peer learning (O'Donnell & King, 1999) and “students as [research and teaching] partners” (Cook‐Sather et al., 2014).Taking hands‐on experiences into the field can further add to students' learning by breaking down the artificial barriers between disciplines (Behrendt & Franklin, 2014; Durrant & Hartman, 2015; Geange et al., 2021). By providing our students the opportunity to iteratively model the scientific process, while explicitly developing both “soft” and “hard” scientific skills, we provide a unique educational experience that yields professional development as well as rich content delivery.

We aim our course at early undergraduate students and seek to position our students as active “researchers,” as well as students, which allows us to model and shift social identities from “students” to “scientists” (Dennett, 1989). FSFE thus provides a unique educational experience that leads students through an intensive, structured reflective process enabling them to explore their own insights as researchers and peers, yielding richness in both professional development and content delivery. Moreover, inspired by the Organization for Field Studies field courses (https://tropicalstudies.org/), FSFE has a flexibility to work across diverse ecological and environmental biology disciplines and ecosystems. With a broad base of contributing experts and specialists, we have run FSFE in alpine and tropical ecosystems in Australia and in tropical systems in Singapore and Malaysia. Each iteration of FSFE covers the same theoretical principles and scientific concepts but is tailored to location‐specific contexts in terms of ecological drivers and locally relevant aspects of protected area management, conservation, and climate change.

## 2 A PRACTICAL OUTLINE OF THE FSFE FIELD‐TEACHING MODEL

**FIGURE 1:**
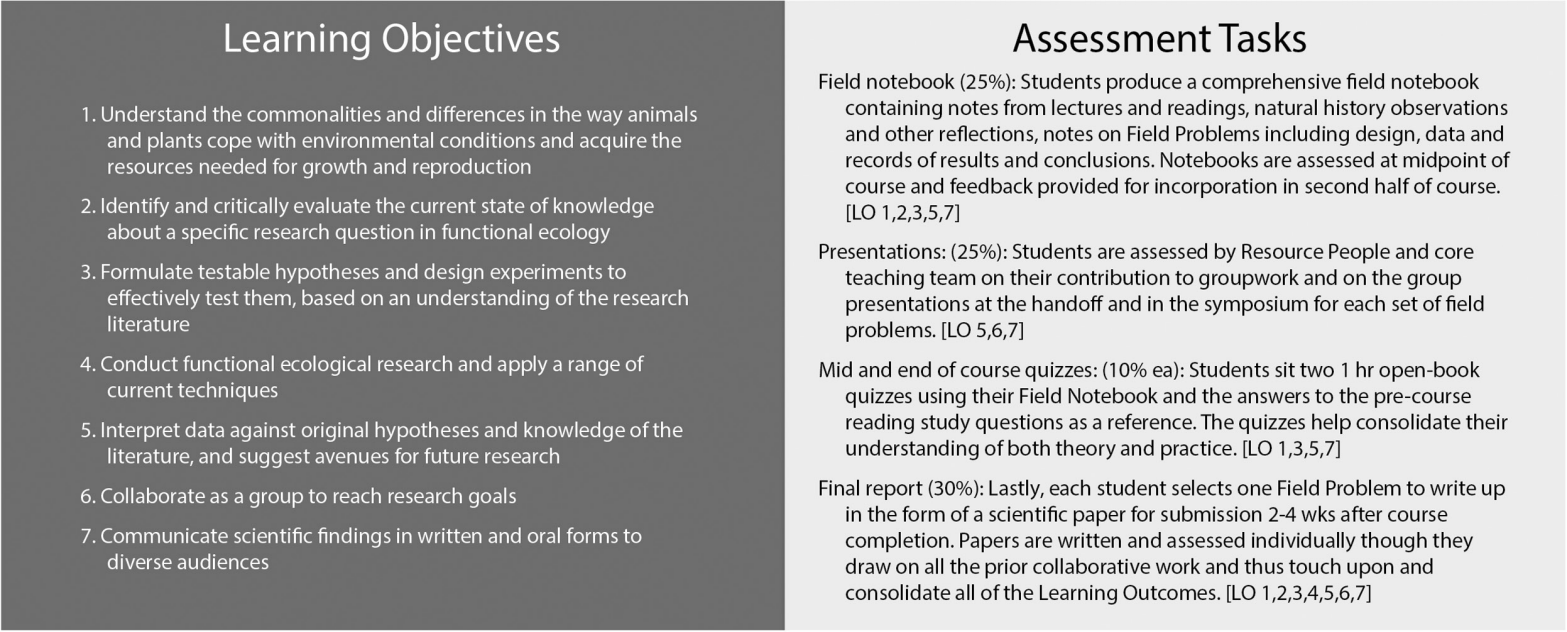
Students are presented with the Learning Outcomes of our course from the outset and Assessment Tasks are aligned to enforce these outcomes. The majority of these are completed during the intensive field component with well‐timed feedback so students can reflect on their work and maximize the value of our iterative model.

The pedagogical underpinnings of the FSFE curriculum include achievable learning outcomes aligned with authentic assessment tasks (Biggs & Tang, 2011; Figure 1). The course's theoretical base lies in cognitive apprenticeship (Brown et al., 1989): through modeling, coaching, scaffolding, articulation, reflection, and exploration (Collins et al., 1989; Dennen, 2004; Enkenberg, 2001), students are “apprenticed” into authentic scientific research practices by the teaching team who explicitly model their expert knowledge and skills in the context of specific learning activities and social collaboration as researchers. We also apply rapid prototyping, whereby scaled‐down processes allow faster design, development, evaluation, and improvement cycles (Dow & Klemmer, 2011; Garrard et al., 2017).

**FIGURE 2:**
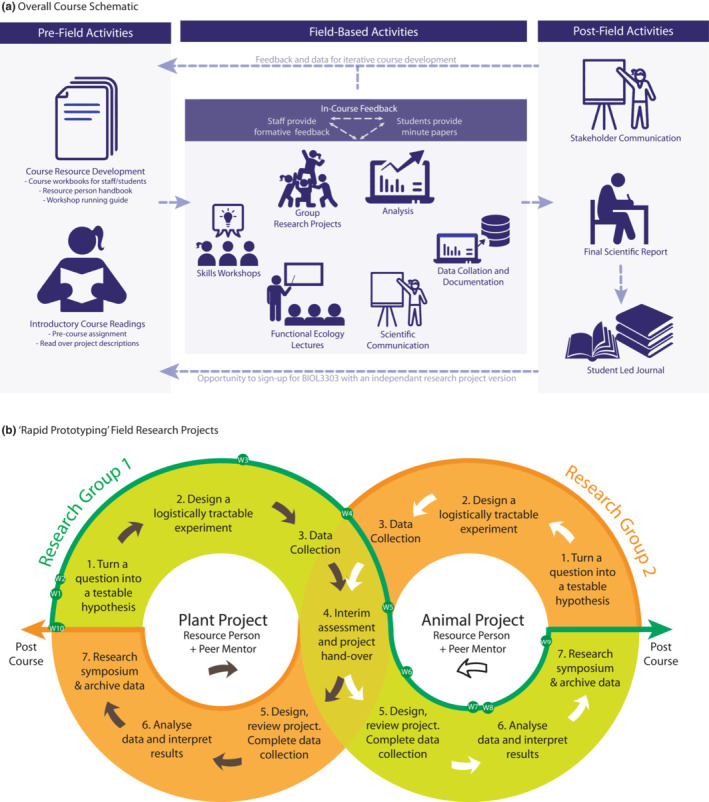
(a) A schematic of overall course structure before, during and after the course. Background content is delivered before the course, the field component aligns skills workshops with phases of the students' research project, and writing follows the field intensive. (b) Illustration of how the students initiate and transfer their projects in each week of the course, showing where each phase of the scientific process applies and where workshops are delivered. Note that workshop 10, Peer Mentoring, is only for the advanced (3000 level students).

With active learning a core focus of FSFE, we deliver just 4 lectures that reinforce relevant theory and 10 workshops that present key skills and concepts (Figure 2). These learning activities are all carefully scheduled to meet students’ immediate needs as they develop their projects, acquire data, and then interpret and present their findings (Figure 2b). Students communicate and refine hypotheses and findings, culminating in a final symposium to which relevant local stakeholders are invited. After the field trip, each student writes a report in the format of a scientific paper, taking time to delve deeper into the literature, and cement their learning. Where rigor and quality are sufficient, students are invited to submit their papers in our open‐access, student‐led journal, where papers are peer reviewed before publication (see below).

From the outset we designed a companion advanced version of the course to accommodate a small number of later year students (~1:3 in comparison to the 2nd year version), including students who had completed the intermediate‐level version. Since 2016, therefore, FSFE has been delivered simultaneously for 2nd‐ and 3rd‐year undergraduates at intermediate and advanced levels, respectively.

**FIGURE 3:**
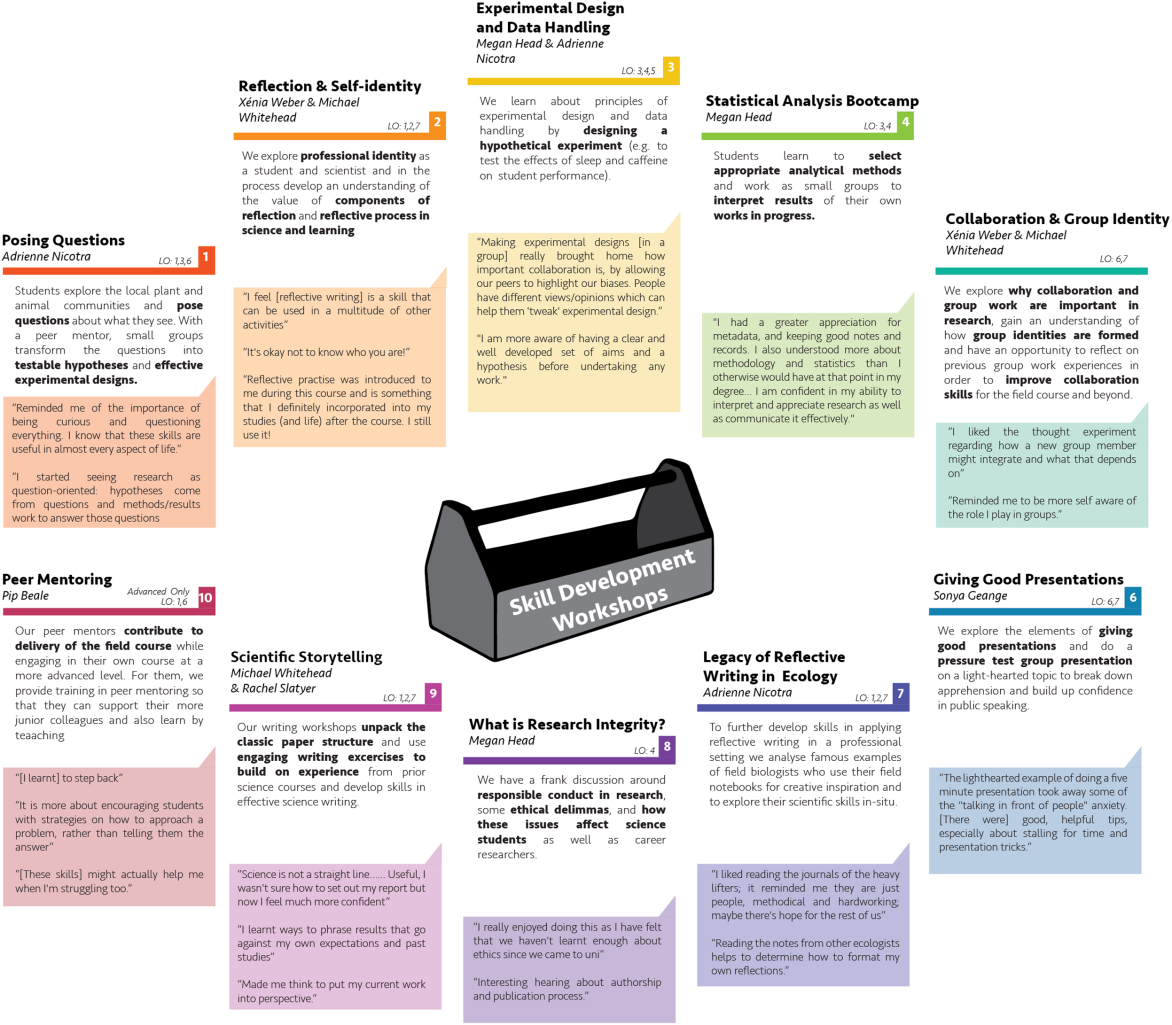
A series of workshops, timed to provide skills at key points in the research process, supports the learning objectives. Quotes drawn from reflective 1‐min paper evaluations conducted at the end of the workshops demonstrate what the students learn from each.

Advanced‐level students carry out independent research projects developed in consultation with a specialist, and participate in progressive skill development through parallel advanced workshops. Importantly, advanced students are trained to be peer mentors (Figure 3, Workshop 10) for intermediate student groups, which provides an enriched learning experience for both (Dolan & Johnson, 2009). The detailed course descriptions that follow are based on the intermediate version of the course.

### 2.1 Teaching team and specialists

Crucial to the success of the FSFE model are the teaching staff. These comprise two groups—the core teaching and technical team responsible for curriculum, workshop delivery, student pastoral care, planning, and logistics; and the transient group of specialists, who we refer to as Resource People.

The heart of FSFE is a series of miniature research projects developed by students from their initial exploration of the field environment and supported by the core teaching team and the specialists from diverse ecological disciplines. These specialists assist, coach, model, and advise but do not determine the direction of the research. As our focus is on ecology fundamentals, only a few of our specialists need detailed knowledge of local areas and species. Importantly, the specialists are integrated into the learning as people: as well as their knowledge and teaching, our specialists share their individual perspectives, career, and life experiences in an initial faculty symposium, contributing to our teaching focus on social and professional identity. This innately human and social perspective helps counter the psychological challenges faced by students as they encounter new concepts, environments, and group‐dynamics when working in the field, especially in more remote settings (Cotton & Cotton, 2009). Like Goodenough et al. (2014), we have observed that excitement and novelty enhance learning outcomes when students are very well supported.

Overall, some 41 staff have participated, with 6 contributing to three or more iterations, and the same senior academic (course convenor) leading all 7 iterations to date. We recruit the specialists from diverse disciplines, balancing the relative expertise in animals and plants. To enable more researchers to experience the benefits of field‐based teaching (Geange et al., 2021), we actively recruit early career researchers into both the core teaching team and as specialists, including Honors or PhD candidates. We embrace high turnover of the specialists as a strength. Past FSFE students are especially welcome.

Most teaching staff have come from our home institution—the Australian National University, Research School of Biology—but we have been privileged to welcome local experts in Far North Queensland, Singapore and Malaysia. Although almost all had some previous university teaching experience, few had previously taught on field courses. All staff are therefore given professional training in field teaching before the course, and constructive support and feedback during the course. Structured evaluations for all staff, in addition to the student evaluations, ensure the core teaching team can act on suggestions from these successive cohorts of specialists, and peer‐to‐peer mentoring often continues well beyond the course duration.

### 2.2 Preparing for the field trip

A month or two before departure, students attend a course induction and Q&A session. They then answer a set of study questions based on precourse readings that focus their attention on key ecological principles along with course‐specific knowledge. Submission of the written responses is a prerequisite for course attendance, though the answers are not graded. For the students, this exercise also provides them with a reference resource during the course, which can be used in the two open‐book quizzes.

In preparation for each course, the teaching team and specialists consider study species well in advance, focusing on those we can reliably, legally and ethically investigate in high enough numbers to yield effective sample sizes, given seasonal and weather constraints. To date, projects have focused on plants, insects, reptiles, and birds, all with requisite scientific licenses. On site, we highlight unique or rare flora and fauna in their ecological contexts, and supplement the research projects with appropriate local highlights (e.g., spotlighting for nocturnal arboreal mammals, talks from local land managers, hikes exploring different habitats).

Before the course, each of the specialist contributors and most or all members of the core teaching team prepare a “Field Problem Abstract” that poses a problem or broad unknown in animal or plant functional ecology—one that interests the specialist and is a genuine open scientific question. Designing projects that can yield a novel discovery and be completed in 4 days is obviously a challenge. The experienced teaching staff work with the specialists to ensure projects are achievable. Publications in our student‐led journal provide examples of what has worked in the past (https://studentjournals.anu.edu.au/index.php/fse). Students are provided with the compiled Field Problem Abstract Book and an accompanying set of project‐specific background readings before the course begins. They are encouraged to explore the abstracts but not expected to do any Field Problem specific readings before departure.

### 2.3 Field research projects developed on site

Throughout the course, the teaching team pays special attention not only to the curriculum but also the students’ mental, social, and physical well‐being. For example, we build the students' sense of belonging in the first few days by having only the core teaching staff present, before subsequently broadening the group and welcoming specialists to join us as we move into the Field Problems component of the course. As an ice‐breaker, and to ground students' understanding of multi‐disciplinarity and complementary teamwork from the start, we begin with an exercise in which students sort themselves in a line that represents a continuum of their relative interest in plants and animals, in molecular versus landscape perspectives, and their relative confidence with statistics. The exercise of mingling among the group and learning how their position varies along different axes is an excellent way to meet one another. The teaching team then use the outcomes to allocate students to research project groups that maximize diversity of existing skills and interests.

On the first day of the course, guided and mentored by the core teaching team, the students investigate the local ecosystem. Through walks and the Posing Questions exercise, they begin formulating ecological questions and developing testable hypotheses based on their observations (Figures 2b and 3, Workshop 1). The following day, the students meet the specialists and learn of their group/field problem allocation (each member of the core teaching team also runs a Field Problem). In this first stage of the process (Figure 2b), each student group works intensively with the relevant specialist to shape a question and hypothesis, and then to design an experiment to test that hypothesis (Figure 2b, Steps 1 to 3).

Crucially, the teaching team supports students to frame questions that consider fundamental concepts in functional ecology, can be effectively executed in the field, and generate data that can be analyzed at an appropriate statistical level, whatever our location. Students are directed to methods resources (e.g., Prometheus Protocols), but must consider the realities of returns, risks, and trade‐offs when developing their methods. Approaches range from the simple (e.g., counts of chosen species or measurements of morphological and physical properties) to more advanced (e.g., physiological assays, such as estimating metabolic rates or biochemical constituents). They learn the relative merits of more sophisticated equipment (e.g., leaf gas exchange systems, animal metabolic systems) that generates data at a finer scale but can be difficult to transport and operate in field settings. They discover when a larger sample size might be obtainable using simple, highly reliable equipment (e.g., rulers, binoculars, scales). In so doing, we have enabled students to learn cutting‐edge techniques and use high‐tech equipment in the field. Each student group then initiates their project and conducts 1.5 days of research, before handing the project to a new group (Figure 2b, Step 4).

The handover is one of most unusual and important elements of our course design. At the halfway point of each project, the students swap projects with another group. This handover involves each group articulating their project's objectives and hypothesis, and the rationale behind their experiment. Each group also hands over a detailed methods document, a dataset—complete with meta‐data—and a dot‐point summary of the results to date, along with any useful resources (e.g., relevant journal papers or analytical tools), and their suggestions for the next phase of the project. Specialists support the handover process and ensure the students' research practices meet modern expectations of data archiving and openness (e.g., the FAIR principles described by Wilkinson et al., 2016). Having such a comprehensive hand‐over process requires all students to reflect on what they have accomplished and tests the data‐handling and communication skills of both “senders” and “receivers.” As students repeat the process during the next research cycle, students can learn from their prior experience how to better facilitate data sharing and learn how to adapt to new collaborations.

Following the handover, each receiving group then takes on board the advice they have received and decides how to progress the received project: in a given handover session we routinely see a full gambit of possible outcomes, from continuing the project unchanged to build a stronger dataset to taking an entirely new experimental approach. After another 1.5–2 days of research, students analyze and interpret their data (Figure 2b, steps 5 and 6). Students then present a ~10‐min conference‐style talk on the entire project, including the initial group's input (Figure 2b, step 7). Lastly, the students archive their data, including meta‐data, detailed methods, and photos, which ensures that all these resources are available for the write‐up phase, while simultaneously teaching a fundamental principle of modern science.

This whole cycle, from project development to handover and project completion, is repeated for a different set of field problems in the second week of the course. As all students are now more familiar with the scientific research process, the second‐round groups tend to be more effective and focused. As the students' progress through the rapid prototyping cycle (Figure 2b), they are continuously prompted to reflect on and develop their skills in collaborative research, including project design and execution, data analysis and interpretation, as well as the oral and written presentation of results.

While this model may appear complex, our aim is something that in practice flows elegantly, an example of cognitive apprenticeship strategies in practice. The short‐term iterative nature of the rapid prototyping encourages quick design, development, and execution. This maintains a high level of engagement and novelty, encourages students to focus on the fundamentals of scientific research practice, and alleviates pressure on students to obtain conclusive results. Supported by workshops on reflective practice and reflective journals as assessed tasks (see below), the process also explicitly invokes reflective evaluation, consolidation, and improvement cycles (Finlay, 2008; Hubbs & Brand, 2005; Kember et al., 2008; Kolb, 1984; Temponi, 2005).

On the final day of the field course, we revisit all the projects that were conducted, and students are asked to reframe one of their projects in the format of rapid‐fire presentation (3 min) aimed at a broad stakeholder audience. Relevant local stakeholders (e.g., land managers, conservation practitioners, tourism operators) are invited to attend and hear what the students have learned as well as to provide them with feedback on their ability to communicate their work to a lay or stakeholder audience. This final presentation is voluntary and not assessed, but almost invariably all the students engage with the exercise in some way and find the presentations a fitting way to celebrate their accomplishments.

### 2.4 In‐field workshops provide an explicit focus on skills development

Skills workshops are a key element of the teaching in FSFE (Figure 3). In addition to the usual scientific process skills, we explicitly name and build “soft” skills—interpersonal strengths, communication, emotional intelligence, reasoning, and problem‐solving skills—that are highly sought by employers in any field (Graduate Careers Australia, 2016; Laker & Powell, 2011; Mauchline et al., 2013; Peasland et al., 2019). By making this part of the course explicit, we find that students have a better appreciation of why we include the workshops and a greater sense of ownership of their learning (Stokes et al., 2011). The workshops are mandatory and one hour long, with most held in the late‐afternoon before students have free time and dinner or occasionally in the evening, after dinner. The workshops are structured around clear objectives (Beckmann et al., 2017), interactive engagement, and summary handouts for students. Regularly updated facilitator handbooks, slide presentations, optional handouts, and relevant equipment enables facilitators to deliver to the same high standards even if they are new to the teaching team. Students provide immediate postworkshop feedback via 1‐minute papers (Stead, 2005), which both provides an opportunity for students to reflect and consolidate their learning and gives staff feedback for continuous refinement of content and delivery.

Skills workshops center on helping students unpack the scientific process (Figure 3). An initial “Posing Questions” workshop (W1) familiarizes students with the local flora and fauna and helps them convert observations and curious questions into testable hypotheses. When each student group has framed its hypothesis, we consider experimental design and data handling (W3). Focused thinking about applied statistics occurs near the end of their first Field Problem (W4). As students prepare their first of several oral presentations, W6 pairs public speaking skills with light‐hearted improvization activities. Toward the end of the course, science writing skills are explored (W9). Finally, we dedicate a session for considering research integrity, moving beyond the normal lectures admonishing plagiarism and instead introducing students to the complexity of scientific authorship, ethical considerations around research and data handling, and the codes of practice that inform professional research (W8). For most students, this first exposure to ethical practice beyond the issue of plagiarism has proved a revelation.

Additional workshops focus on building a researcher identity and developing skills in collaboration and reflective practice; these are key course goals related to the cognitive apprenticeship model (Figure 3). These innovative workshops build on affective learning as a strong component of field courses (Beckmann et al., 2017; Boyle et al., 2007). At the start of the course, we explore the concepts of personal reflective practice and the “social identity approach” (Haslam, 2004) in relation to behavior within and between teams of collaborating researchers (W2). Through regular entries in the field notebooks, students reflect on their participation and the course as part of an experiential learning cycle (Kolb, 1984; Moon, 1999) with a view to gaining insight into themselves as learners and scientists. After the first project, students are also able to reflect on their own and their team's challenges and strengths as collaborators, so we extend our discussion by exploring how students and research scientists, and ecologists in particular, build self‐identity and complementary teamwork skills (W5). A focus on reflection as part of research practice comes next (W7). Knowing that students might conflate reflective journals with simple diaries, we explore the field journals of notable naturalists and ecologists to demonstrate how reflection on field observations and notes have contributed historically to the development of ecological theories. This helps students see their Field Notebook assessment task more holistically.

### 2.5 After the trip

By the end of the course, each student has participated in four different projects (two on animals and two on plants), one of which they select to write up as their final paper due 2–4 weeks after return. The write‐up draws on the methods, data, and presentation materials that were put in our archive during the course. This both models a key element of contemporary science and gives the students maximum flexibility in writing up their final paper (Gallagher et al., 2021; Parker et al., 2016). The final papers are written individually, in the style of a research paper, and follow the format of our student‐led journal.

**FIGURE 4:**
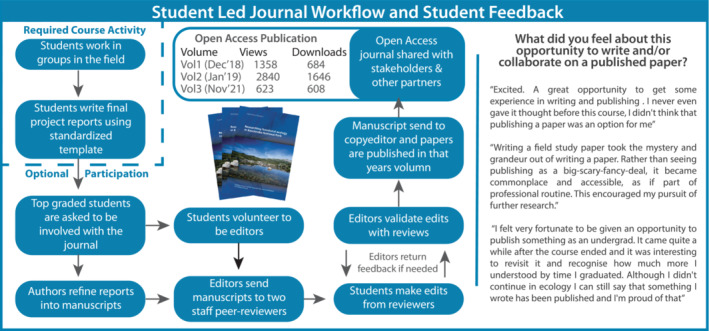
Field Studies in Ecology is an open‐access, student‐edited, peer‐reviewed journal that makes student research accessible for subsequent students, stakeholders, and the broader community while also giving the students genuine experience of the process of scientific writing, review and publication. Downloads data current as of Aug, 2022.

Throughout FSFE, we emphasize researchers' responsibility to communicate and ideally publish their findings to maximize potential impact (US National Research Council, 2003). Student‐led undergraduate journals are relatively rare, especially in science, yet are known to provide particularly powerful learning, especially if peer review experiences are included (Guilford, 2001; Uigín et al., 2015). From the first iteration of FSFE, we inaugurated an open access journal “Field Studies in Ecology” (Figure 4; see Appendix S1 for more detail). Students who achieved a “Distinction” (~>70% or a B) for their final report can choose to submit a manuscript for peer review. The expectations for these junior authors are high: they need to show a substantive understanding of relevant discipline knowledge, critical thinking, and data analysis and synthesis, and scientific writing, alongside thoughtful responses to feedback from the expert peer reviewers. The journal's editors are also FSFE students, selected for each volume through expressions of interest along with academic performance. Mentored by academics and professional editors, these student editors take on significant responsibilities in the peer review and publication process, including managing all student authors and academic peer reviewers selected from the current and former specialists, colleagues in our Research School, and where relevant, external researchers.

Three volumes of “Field Studies in Ecology” have been published to date (see https://studentjournals.anu.edu.au/index.php/fse). Volumes 1 and 2, respectively cover the 2015 and 2016 courses at Kosciuszko National Park (Hazell‐Pickering et al., 2019; Zurcher et al., 2017). Volume 3 includes research from 2017 at the Daintree Rainforest (Cape Tribulation, Queensland) and 2018 at the Bukit Timah Nature Reserve in Singapore (Harris et al., 2021). Volume 4 is in preparation. The journal makes the FSFE student data available to stakeholders—government agencies, land managers, industry professionals. Analytics show all volumes are viewed and downloaded regularly (Figure 4).

## 3 ASSESSING THE EFFECTIVENESS OF THE FSFE MODEL

**FIGURE 5:**
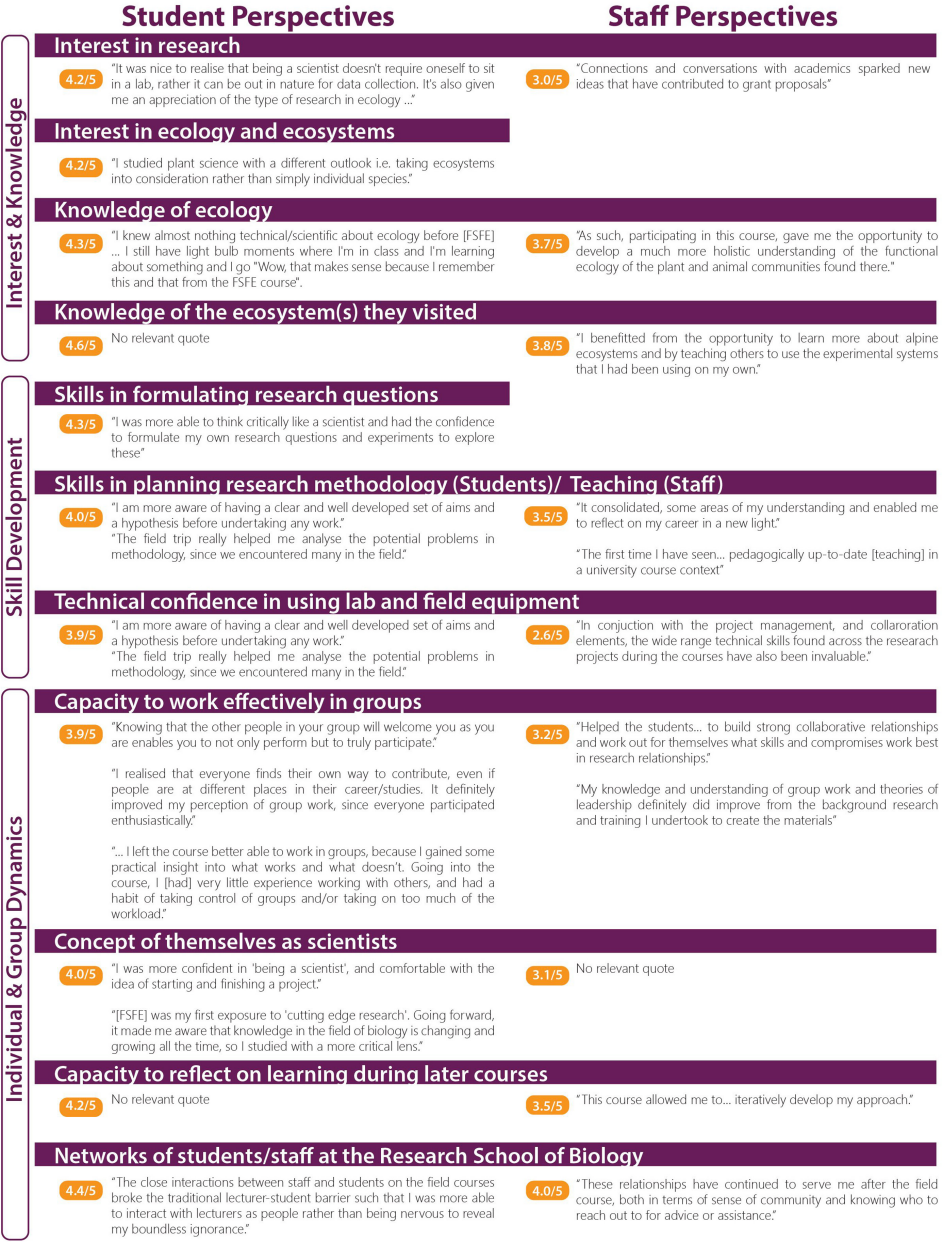
Responses of students (left column) and staff (right) to a retrospective survey of perceptions of the impact of FSFE. The survey questions used a Likert‐scale ranging from 1 (not at all) to 5 (very much). Mean ratings above 3/5 were considered to indicate an increase in the relevant sphere. Open ended comments from the survey are included to illustrate impacts. Blank spaces on the staff side indicate that a question was not asked of the staff.

A continuous improvement cycle (Temponi, 2005) has been a feature of all facets of the course since the first iteration. In 2020, we sought evidence about the long‐term impacts of FSFE in three ways. First, we surveyed all students for whom we had current email details (*n* = 108) and staff (*n* ~ 40) who had participated in the course using an online survey that included Likert‐scale questions on FSFE's impact on the students'/staff areas of interest, knowledge and skills, and open‐ended questions on perceptions of the impact of FSFE overall. Of these, 43 students and 21 staff replied and their results are summarized as mean Likert‐scale scores and percentages. Second, we collated a sample of 85 paired reflective writing entries from field notebooks, written in the middle and at the end of the course, and assessed the relative development of reflective practice. Entries were assessed using ordinal logistic regression in R (MASS) across the four attributes associated with effective reflective writing—descriptive detail, emotive engagement, critical reflection, and meta‐reflection—using the assessment rubric that we provide to the students (Kember et al., 2008; Moon, 1999). Third, we compared the academic outcomes of our students using a paired student design (*n* = 105 pairs) where students are compared using GLMM in R to a student from the same degree and who achieved the same grade in the prerequisite first year course who did not complete FSFE. The survey results are summarized in Figure 5 with representative quotes and as answers to the four questions below (further detail on methods and analyses is available in Appendix S2 and survey questions are presented in Appendix S3).

### 3.1 How has FSFE influenced students' study and career paths?

Although FSFE aims to deliver a high standard of research skills and opportunities, it was not designed to only serve students seeking research careers in ecology. Our quantitative analysis showed that students who took FSFE (either as a second year, third year or both) were significantly more likely to complete their degree (*χ* ^2^ = 26.251, *p* < .001, noncompletion of FSFE students = 3.5% versus noncompletion of non‐FSFE students = 17.6%, see Appendix S1). However, we are unable to determine whether this is a consequence of taking FSFE or due to an intrinsic property of the students who take FSFE (i.e., students that choose to take a course like FSFE are more likely to finish their degree). In the retrospective student survey, respondents reported substantial impacts of their FSFE experience on their subsequent studies and interests (Figure 5). For example, among the students who responded, FSFE had motivated some to take more biology and ecology courses in their science degree than they had originally planned, and many acknowledged FSFE as having motivated them to pursue research careers. Indeed, most (70%) of the 25 student survey respondents who had graduated since taking FSFE had continued into research (Honors, Masters programs, Doctor of Philosophy) or further study (Doctor of Medicine degrees), and others were working in related fields—science communication, government science policy, or nonuniversity ecology research. However, this did not differ significantly from the paired students who did not complete FSES (Appendix S1). We note that our results likely reflect some product of self‐selection, as students who continued in science related areas may have been more likely to respond. Nonetheless, most (76%) reported that FSFE had given them confidence to identify themselves as scientists, regardless of their subsequent study and career paths.

Almost all the respondents to the student survey reported that they had approached learning and community‐building differently after FSFE. The novel settings, the student‐led approach, and the opportunities for high‐quality interactions with staff stood out for many as specific features of the course. Many reported that FSFE provided their first experience of “real science”—authentic exploration and discovery. Although our quantitative comparison of their outcomes in terms of GPA did not reflect a statistically significant impact given the small sample size (see Appendix S1), students reported that the improved confidence and skills they felt they had gained during the course positively impacted their subsequent academic performance.

Students described how being supported through the scientific process in FSFE had enabled them to think more critically, assimilate information with greater ease, and develop specific scientific/academic skills that benefited their future study. In open‐ended responses, many described the lasting impact of FSFE: these students felt the course had affected them in ways that still permeated both their personal and academic lives, stimulating memories and reflections years later.

### 3.2 Do FSFE students develop skills in research and scientific thinking?

Student survey respondents reported notable increase (mean Likert‐score 4.6/5) not only in their *knowledge* of ecology and the ecosystems they visited but also in their *interest* in ecology and ecosystems, and in research overall. Some two thirds reported notable increases in their ongoing skills (mean Likert‐score 4.3/5) in formulating research questions and planning research methodology after FSFE, while a similar proportion noted increased technical confidence in using lab and field equipment. The knowledge sharing in FSFE often occurs “just in time” that is, in relation to genuine curiosity and a “need to know.” Our students reported that they could assimilate a much greater amount of complex content in this learning context compared to the equivalent taught in packaged lectures in a campus‐based delivery model.

### 3.3 Do FSFE students develop skills in reflective practice?

As educators we wanted to know whether FSFE was improving students' capacity for reflective practice. As we had provided both training and written mid‐course feedback on each students' individual reflections, we hypothesized a comparison would reveal that students' reflective proficiency and competencies would improve over the course. Students scored consistently high on descriptive detail in their reflective writing from the beginning of each course, which is expected given the students were motivated and this is the most basic attribute of reflective writing (Moon, 1999). By contrast, by the end of the course scores had significantly increased for increasingly sophisticated and more effective reflective practice including emotive engagement with their experiences (*p* = .01), ability to critically reflect, evaluate and analyze (*p* < .001), and ability to reflect on the value of reflection (meta‐reflection, *p* < .001). Overall, and especially in students who attended both intermediate and advanced iterations, the journals demonstrated a clear shift from “reflection *on* action” to “reflection *in* action” which Schon (1983) considered the core of “professional artistry,” and Finlay (2008) described as indicating an expert who acts “both intuitively and creatively [as they] revise, modify and refine their expertise.” This analysis of the reflective journal data were supported by the student survey findings: for example, most respondents (76%) reported a greater capacity to reflect on their own learning in their subsequent university courses after completing FSFE (mean Likert‐score 4.2/5).

### 3.4 Do FSFE students develop teamwork skills?

Another key focus of FSFE is collaborative teamwork. In each iteration, we have observed tangible improvements in teamwork. For example, the teaching team regularly observes that individuals become better at drawing on the diverse skills and capabilities of their peers as well as of the specialists, and at sharing responsibilities, outcomes, and discoveries. The reflective culture adds to this by facilitating a greater awareness and tolerance of their own and their peers' limitations.

In the student survey, most respondents (83%) reported that FSFE had increased their capacity to work effectively in groups. Responses to open‐ended questions showed that these FSFE participants felt that being supported through the scientific process had enabled them to think more critically, assimilate new information with greater ease, and develop specific scientific/academic skills that benefited their future work and study. Most respondents (83%) also reported that FSFE had initiated a notable growth in their networks of peers and staff.

### 3.5 FSFE staff evaluation of the teaching model

Lastly, we assessed the feedback from 21 staff who responded to the 2020 survey for their insights on the teaching model for students and for their own professional development (Figure 5 and quotes therein). The staff reported that FSFE students benefited from the course's applied, immersive nature: needing to be realistic in data collection and project design/management had given the students a “real” experience of practicing science. Irrespective of previous teaching experience, all staff respondents also reported that participating in FSFE had improved their teaching skills, especially their capacity for reflecting on their teaching practice. Staff also commonly reported that teaching in FSFE had increased their technical confidence and broader knowledge of ecology, enlarged their professional networks and research collaborations, increased the number of students seeking supervision in research degrees, and had provided unique opportunities to give and receive mentorship.

## 4 CLOSING REMARKS

Our evaluation of 5 years of FSFE has shown a great array of positive outcomes including reports of increased self‐efficacy, learning gains, confidence, collaboration skills, research interest, and more among our students. We have found that pairing ecological content and skills development workshops with our rapid prototyping of research projects under “apprenticeship” to specialists has proven highly effective. Explicitly weaving concepts of social identity and reflective practice into the course, and a commitment to teaching complex “soft” skills like collaboration, reflective practice, and teamwork has had clear benefits. The measured shift from reflection “*on*” action to reflection “*in*” action indicates individuals more capable of recognizing, and engaging with the diverse skills and capabilities of their cohort. Further, as we have continuously evaluated and fine‐tuned our model, FSFE has become an increasingly more effective and novel teaching tool that delivers major, positive impacts on student academic and professional development.

FSFE is a vehicle for learning, teaching, and practicing authentic contemporary science, including data sharing, peer review, and open access publishing. We hope we have demonstrated how the FSFE field course model provides an outstanding vehicle for research‐led education that finds and nurtures talent, actively engages with stakeholders beyond the university, and fosters collaborative and reflective practice that is preparing students to address the pressing real‐world challenges. Imbued with the intentional perspective that we all share a scientist identity, we aim to facilitate a gentle but enduring identity transition from “we are a group of students and academics” to “we are all scientists creating knowledge together.”

We hope that our exploration of how the FSFE course functions as an evolving form provides inspiration for development of other field courses. More detail on course components, schedules, abstract books, and workshop are available at the course webpage: https://www.ecologyfieldstudies.org/. Through field courses, our students gain authentic research experience and come to appreciate both what the skillset of a scientist is and what the value of those skills is in a diverse array of successful careers. Maintaining field courses in an undergraduate curriculum can be challenging due to the logistical constraints and costs, but these courses are so important to developing skills that improve graduate employability that they are crucial to ensuring well‐rounded undergraduate experiences (Mauchline et al., 2013).

## AUTHOR CONTRIBUTIONS

**Adrienne Nicotra:** Conceptualization (lead); data curation (supporting); formal analysis (supporting); funding acquisition (lead); investigation (lead); methodology (equal); project administration (lead); resources (lead); supervision (lead); validation (supporting); writing – original draft (equal); writing – review and editing (equal). **Sonya Rita Geange:** Conceptualization (equal); data curation (equal); investigation (equal); methodology (equal); resources (equal); supervision (equal); visualization (equal); writing – original draft (equal); writing – review and editing (equal). **Nur Bahar:** Methodology (equal); project administration (equal); resources (equal); supervision (equal); writing – original draft (equal); writing – review and editing (equal). **Hannah Carle:** Investigation (equal); writing – original draft (equal); writing – review and editing (equal). **Alexandra Catling:** Investigation (equal); writing – original draft (equal); writing – review and editing (equal). **Andres Garcia:** Investigation (equal); writing – original draft (equal); writing – review and editing (equal). **Rosalie Harris:** Investigation (equal); writing – original draft (equal); writing – review and editing (equal). **Megan L. Head:** Conceptualization (equal); investigation (equal); methodology (equal); supervision (equal); writing – original draft (equal); writing – review and editing (equal). **Marvin Jin:** Investigation (equal); validation (equal); writing – original draft (equal); writing – review and editing (equal). **Michael R. Whitehead:** Conceptualization (equal); data curation (equal); formal analysis (equal); investigation (equal); supervision (equal); writing – original draft (equal); writing – review and editing (equal). **Hannah Zurcher:** Investigation (equal); writing – original draft (equal); writing – review and editing (equal). **Elizabeth Beckmann:** Conceptualization (equal); data curation (equal); formal analysis (equal); investigation (equal); project administration (equal); resources (equal); supervision (equal); validation (equal); writing – original draft (equal); writing – review and editing (equal).

## CONFLICT OF INTEREST

The authors have no conflicts of interest to declare.

## Supporting information


Appendix S1

Appendix S2

Appendix S3

## Data Availability

Data were collected under human research ethics protocols and will be provided in accordance with those protocols on request.
